# Deoxyguanosine kinase deficiency and recurrent spontaneous pneumothorax: a case report

**DOI:** 10.1186/s13256-023-04151-1

**Published:** 2023-09-30

**Authors:** Alice Ramboux, Alain Poncelet, Philippe Clapuyt, Isabelle Scheers, Etienne Sokal, Raymond Reding, Xavier Stephenne

**Affiliations:** 1 Division of Paediatric Gastroenterology and Hepatology, Department of Paediatrics, Cliniques Universitaires Saint-Luc, Université catholique de Louvain, Brussels, Belgium; 2 Division of Cardiothoracic Surgery, Department of Surgery, Cliniques Universitaires Saint-Luc, Université catholique de Louvain, Brussels, Belgium; 3 Division of Paediatric Radiology, Department of Radiology, Cliniques Universitaires Saint-Luc, Université catholique de Louvain, Brussels, Belgium; 4 Division of Paediatric Surgery, Department of Surgery, Cliniques Universitaires Saint-Luc, Université catholique de Louvain, Brussels, Belgium

**Keywords:** Deoxyguanosine kinase deficiency, Mitochondrial DNA depletion syndrome, Mitochondriopathy, Amyotrophy, Recurrent spontaneous pneumothorax, Case report

## Abstract

**Background:**

Deoxyguanosine kinase deficiency is mainly manifested by hepatic and neurological damage, hence it belongs to the hepatocerebral form of mitochondrial deoxyribonucleic acid depletion syndrome. The association between deoxyguanosine kinase deficiency and recurrent spontaneous pneumothorax has not currently been reported.

**Case presentation:**

A 12-year-old Russian boy with deoxyguanosine kinase deficiency, a recipient of a liver transplant with amyotrophy secondary to his mitochondriopathy, presented with recurrent spontaneous bilateral pneumothorax refractory to drainage and surgery.

**Conclusion:**

To our knowledge, this is the first documented case of deoxyguanosine kinase deficiency associated with recurrent spontaneous pneumothorax, which could be considered a late complication of deoxyguanosine kinase deficiency. At this point, this is only an association and further studies and research need to be performed to help confirm the pathogenesis of this association.

## Background

Deoxyguanosine kinase (DGUOK) deficiency is part of hepatocerebral mitochondrial deoxyribonucleic acid (DNA) depletion syndrome (MDS). MDS represents a broad spectrum of autosomal recessive diseases characterized by reduced copy number of mitochondrial DNA (mtDNA), resulting in mitochondrial dysfunction and insufficient energy production in the affected tissues and organs [1–5]. The phenotypic presentations of MDS are heterogeneous with a generally early onset. Depending on the affected organs, MDS are classified into four categories: myopathic, encephalomyopatic, hepatocerebral, and neurogastrointestinal forms. Each category results from different nuclear gene mutations [1, 3]. The exact prevalence of MDS or DGUOK deficiency is still unknown. Hepatocerebral manifestations of MDS are the most common and DGUOK deficiency is estimated to account for 15–20% of all cases of mtDNA depletion [4]. DGUOK-related MDS can present in two forms: a multiorgan disease in neonates or an isolated liver disease with infancy or childhood onset [3]. The majority of affected patients develop multisystemic manifestations, commencing in the first weeks of life, such as lactic acidosis, hypoglycemia, hepatic dysfunction, and neurological damage. Later, severe myopathy, developmental delay, and nystagmus may also follow [1–5]. The DGUOK-related MDS affected patients usually die within the first year of life, mainly due to liver failure [1, 2]. Existing data on transplant patients show that the neurologic damage may occur and/or worsen after liver transplantation. Therefore, the decision to perform a liver transplant in these patients remains controversial [2, 4].

Spontaneous pneumothorax is a known complication of several genetic syndromes and is defined by the presence of air in the pleural space originating neither from trauma nor from iatrogenic causes [6]. Recurrent spontaneous pneumothorax has recently been reported in X-linked myotubular myopathy (XLMTM), an inherited myopathy [7, 8]. So far, to the best of our knowledge, there is no existing data describing the association between DGUOK deficiency and recurrent spontaneous pneumothorax.

## Case presentation

**Fig. 1:**
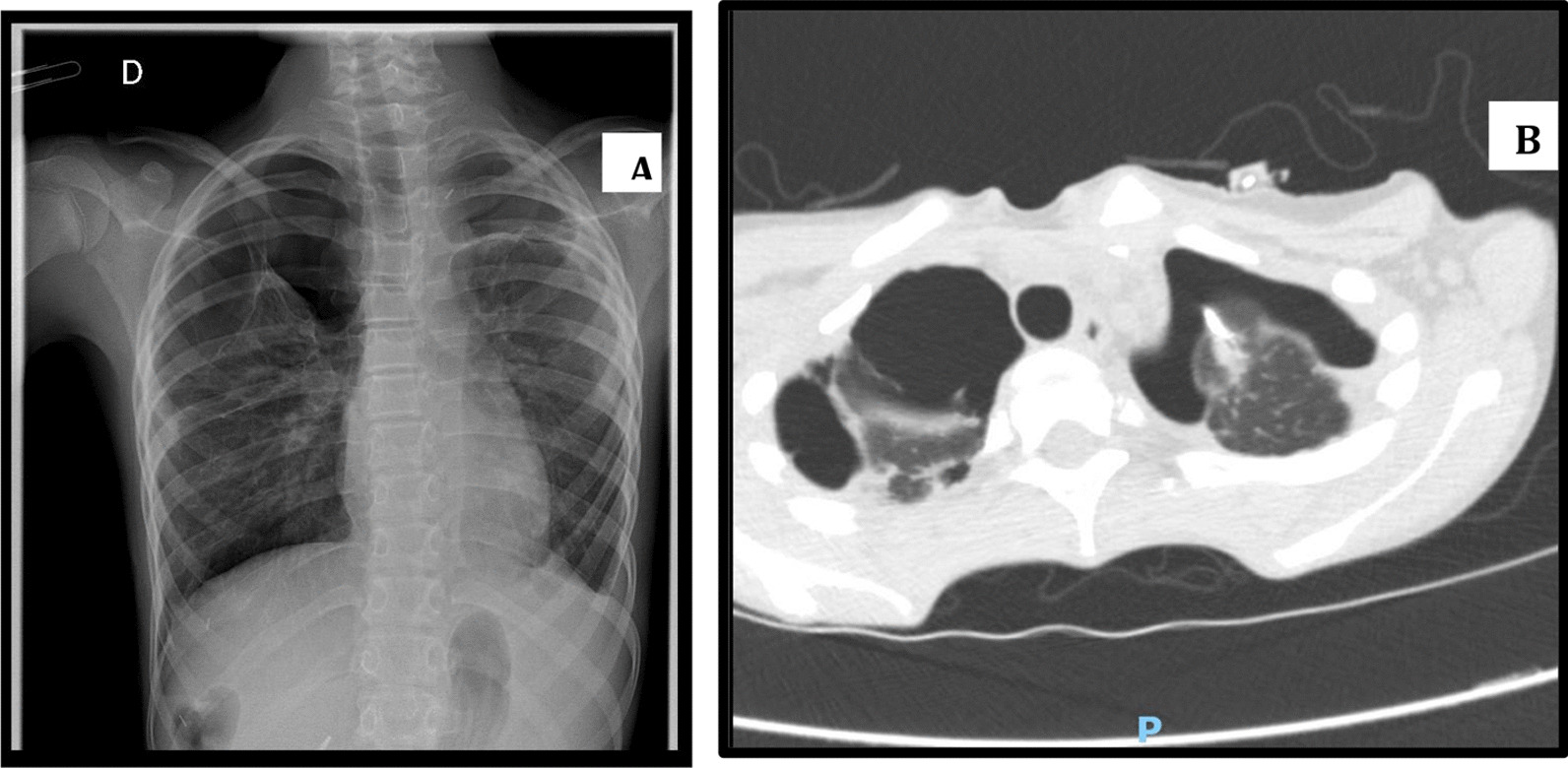
Bilateral pneumothorax in medical imaging. **A** Frontal chest X-ray of the patient showing a bilateral apical pneumothorax. **B** Thoracic computed tomography (CT) scan of the patient. Presence of a bilateral (hydro)pneumothorax with pleural thickening and predominant adhesions on the right

A 12-year-old Russian boy, born to non-consanguineous parents, presented at the age of 2 weeks with hypoglycemia, metabolic acidosis, and neonatal cholestasis rapidly progressing to liver failure and encephalopathy. After exclusion of contraindications, a liver transplant was performed at the age of 11 months, the etiology of liver failure remaining undetermined at that time. Posttransplant follow-up was marked by portal vein stenosis treated by mesorex shunt surgery. Later, at the age of 8 years, a genetic test revealed a homozygous mutation in the first exon of DGUOK (c.3G>A, p.Met1Ile). This is the most common mutation leading to the hepatocerebral form of MDS and isolated liver failure in the Russian population [9]. At the age of 12 years, the patient presented with important amyotrophy and an occasional cough that became chronic. Faced with an intensification of respiratory complaints and a suggestive clinical presentation, a radiological assessment was performed and evidenced a bilateral pneumothorax (Fig. 1A, B). In the space of 4 months, the patient presented with almost a dozen spontaneous pneumothoraces, requiring multiple pleural drainages and an unsuccessful left surgical pleurodesis. The lung biospy revealed emphysema and extensive fibrous pleurisy. The microbiological work-up of the pleural liquid was negative. During the last hospitalization, a chemical pleurodesis was considered but was impossible to perform given the incomplete reexpansion of the lungs; therefore, a Heimlich valve was placed instead. The boy was followed up in ambulatory care with regular chest X-rays to follow the progressive filling of the two pleural cavities apices (which may occur in such circumstances). The remaining thoracic drain was quickly removed during outpatient checks. Currently, the patient is asymptomatic and still presents with a bilateral pleural detachment, which remains stable while the dead space at the left lung apex is starting to fill.

## Discussion

We present the case of a 12-year-old child with DGUOK deficiency having recurrent spontaneous pneumothorax, which, to date, has never been described in the literature as associated with DGUOK deficiency. At this point, this is an association and should be confirmed with other similar cases.

Just over 100 cases have been identified with pathogenic variants in DGUOK [3, 4]. The description of the phenotypic features associated with this pathology is based on these reports, which teach us that the majority of patients with MDS caused by the same mutation as our patient suffered from a neonatal multiorgan illness that manifested as lactic acidosis and hypoglycemia in the first week of life. A few weeks after birth, all infants developed hepatic disease and neurologic dysfunction, such as hypotonia. Later, severe myopathy, developmental delay, and nystagmus developing into opsoclonus may also follow. A minority of affected individuals present initially in infancy or childhood with isolated hepatic disease [1–5]. Liver involvement can cause progressive and usually fatal liver failure within the fist year of life [1, 2]. An important similarity exists between the initial symptoms presented by our patient and the cases described with MDS related to DGUOK. On the other hand, our patient never presented with nystagmus evolving towards opsoclonus. As previously mentioned, most patients affected by DGUOK deficiency die within the first year of life, which could explain why spontaneous pneumothorax in DGUOK deficiency has not yet been reported. Again, the genetic diagnosis was not available at the time of the transplantation.

Our bibliographic research points to the key role of mitochondrial dysfunction in the genesis of pneumothorax and amyotrophy, both presented by our patient.

In physiological states, mitochondria are able to produce large amounts of adenosine triphosphate (ATP) and calcium buffers with low production of reactive oxygen species (ROS). Energy resources and calcium buffers are then transported following local cellular needs via the anterograde and retrograde movement of mitochondria along the axons by motor proteins.

In amyotrophic lateral sclerosis (ALS), mitochondrial ATP production and calcium buffers capacity are reduced, while ROS production is elevated. Alterations in the axonal transport of mitochondria are noted, as well as a depletion of the mitochondrial population at the neuromuscular junction [10]. Alterations in mitochondrial function found in ALS may be similar in DGUOK deficiency. Increased production of ROS in the lung region may contribute to spontaneous pneumothorax in DGUOK deficiency, as well as amyotrophy itself.

In lymphangioleiomyomatosis (LAM), a rare progressive systemic disease characterized by impaired lung function, lung destruction and spontaneous pneumothorax are caused by neoplastic growth of atypical smooth muscle-like LAM cells [11]. In 2019, Abdelwahab *et al.* demonstrated that mitochondrial dysfunction is a key determinant of this orphan disease and provides a novel therapeutic target. Their study details molecular analysis of patient-derived LAM cell lines, allowing the assessment of mitochondrial function and biogenesis to be defined in the pathomechanism of LAM. They highlight an increase of mitochondrial reactive oxygen species (ROS) production, which can cause oxidative injury, critically damage mitochondria of LAM cells, and therefore result in abnormal mitochondrial function. They theorize that drugs that can restore normal mitochondrial function might be able to reduce LAM progression.

We also found spontaneous pneumothorax cases associated with hereditary myopathies in the literature. Carsten *et al.* [8] reported spontaneous pneumothorax in two brothers with XLMTM in adulthood. Yabe *et al.* [7] also described spontaneous pneumothorax in an 8-year-old child with XLMTM.

## Conclusion

To our knowledge, this is the first documented case of DGUOK deficiency associated with spontaneous pneumothorax. The first symptoms presented by our patient are similar to those of the cases described with DGUOK-related MDS. The pneumothorax presented by our patient could be considered as a late consequence of DGUOK deficiency. We suggest a preponderant role of amyotrophy secondary to the disease, as well as an increased production of ROS caused by the alteration of mitochondrial function in the genesis of pneumothorax in our patient. Our case could therefore broaden the phenotypic spectrum of DGUOK mutations. At this point, this is only an association and should be confirmed either through pathogenesis or with other similar cases before this can be ascribed as part of this disease. It would therefore be important for the clinician to be attentive to the pulmonary status in patients with DGUOK deficiency.

## Data Availability

Not applicable.

## Abbreviations

DGUOK: Deoxyguanosine kinase
MDS: Mitochondrial DNA depletion syndromes
mtDNA: Mitochondrial DNA
XLMTM: X-linked myotubular myopathy
ATP: Adenosine triphosphate
ROS: Reactive oxygen species
ALS: Amyotrophic lateral sclerosis
LAM: Lymphangioleiomyomatosis
